# Insights and limits of translational research in critical care medicine

**DOI:** 10.1186/s13613-015-0050-3

**Published:** 2015-04-30

**Authors:** Frédéric Pène, Hafid Ait-Oufella, Fabio Silvio Taccone, Guillaume Monneret, Tarek Sharshar, Fabienne Tamion, Jean-Paul Mira, on behalf of the Commission de Recherche Translationnelle de la Société de Réanimation de Langue Française (SRLF)

**Affiliations:** Service de Réanimation Médicale, Hôpital Cochin, Assistance Publique-Hôpitaux de Paris, 27 Rue du Faubourg Saint-Jacques, 75014 Paris, France; Faculté de Médecine, Université Paris Descartes, 12 Rue de l’Ecole de Médecine, 75006 Paris, France; Service de Réanimation Médicale, Hôpital Saint-Antoine, Assistance Publique-Hôpitaux de Paris, 184 Rue du Faubourg Saint-Antoine, 75012 Paris, France; Faculté de Médecine, Université Pierre-et-Marie-Curie, 27 Rue Chaligny, 75571 Paris, France; Département de Soins Intensifs, Hôpital Erasme, Université Libre de Bruxelles, Route de Lennik 808, 1070 Brussels, Belgium; Laboratoire d’Immunologie Cellulaire, Hôpital Edouard Herriot, Hospices Civils de Lyon, 5 Place d’Arsonval, 69003 Lyon, France; Institut des Sciences Pharmaceutiques et Biologiques, Université Lyon I, 8 Avenue Rockefeller, 69373 Lyon, France; Service de Réanimation, Hôpital Raymond Poincaré, Assistance Publique-Hôpitaux de Paris, 104 Boulevard Raymond Poincaré, 92380 Garches, France; Université de Versailles-Saint Quentin en Yvelines, 55 Avenue de Paris, 78000 Versailles, France; Service de Réanimation Médicale, Hôpital Charles Nicolle, CHU Rouen, 1 Rue de Germont, 76000 Rouen, France; Faculté de Médecine, Institut de Recherche et Innovation Biomédicale (IRIB), 22 Boulevard Gambetta, 76000 Rouen, France

**Keywords:** Critical care medicine, Translational research, Sepsis, Microcirculation, Critical illness-related neuromyopathy

## Abstract

Experimental research has always been the cornerstone of pathophysiological and therapeutic advances in critical care medicine, where clinical observations and basic research mutually fed each other in a so-called translational approach. The objective of this review is to address the different aspects of translational research in the field of critical care medicine. We herein highlighted some demonstrative examples including the animal-to-human approach to study host-pathogen interactions, the human-to-animal approach for sepsis-induced immunosuppression, the still restrictive human approach to study critical illness-related neuromyopathy, and the technological developments to assess the microcirculatory changes in critically ill patients. These examples not only emphasize how translational research resulted in major improvements in the comprehension of the pathophysiology of severe clinical conditions and offered promising perspectives in critical care medicine but also point out the obstacles to translate such achievements into clinical practice.

## Review

Experimental research has always been the cornerstone of pathophysiological and therapeutic advances in medicine. Accurate modeling of human pathology remains challenging but is essential to draw relevant conclusions from experiments. With respect to critical care medicine, the high mortality rate and morbidity imposed by severe acute conditions prompted an intense research activity in which clinical observations and experimental research mutually fed each other in a so-called translational approach. The objective of this review is to address the different aspects of translational research in the field of critical care medicine. We herein highlighted some demonstrative examples of translational research including the animal-to-human approach to study host-pathogen interactions, the human-to-animal approach for sepsis-induced immunosuppression, the human-to-human approach to study critical illness-related neuromyopathy, and the technological developments to assess the microcirculatory changes in critically ill patients. We will not only discuss how the promises of translational research could be fulfilled in the bedside management of critically ill patients but also the obstacles to translate such achievements into clinical practice.

### The foiled promises of host-pathogen interactions: from animals to humans

The discovery of Toll-like receptors (TLRs) and the resultant advances in the understanding of pathophysiology of sepsis represent an excellent example of both complementarities and limits of translational research. The story began in the late nineteenth century when Elie Metchnikoff conducted experiments on the transparent starfish larvae in which he planted pine needles. Using a single optical microscope, he observed a cellular infiltrate that rapidly surrounded the foreign body with vesicles of phagocytosis and described for the first time the cellular immune response against a foreign agent [1]. For his works in innate immunity, mostly performed in invertebrates, Metchnikoff received the Nobel Prize in Medicine and Physiology in 1908. However, despite the intense research that was prompted by the initial observation, the human receptors for pathogen molecular patterns such as the receptor to endotoxin remained a mystery for nine decades. The identification of TLRs as pattern-recognition receptors (PRRs) for conserved structures of bacteria and viruses and the acknowledgement of their major roles in the inflammatory response were made possible thanks to basic research. Indeed, a receptor protein whose structure is very similar to TLRs was first described in tobacco plants [2]. Two years after, Jules Hoffmann and his group reported similar proteins in the *Drosophila* fruit fly and showed that the mutation of a receptor called Toll inhibited the production of an antifungal peptide and increased susceptibility to fungal infection, a discovery that was awarded by the 2011 Nobel Prize for Medicine and Physiology. [3]. Williams et al. also reported that *Drosophila* larvae carrying a mutation on a protein of the Toll signaling pathway (18-wheeler) had an increased susceptibility to bacterial infections [4]. Based on these experimental results, Medzhitov and Janeway cloned the first TLR in humans. They confirmed the link between TLRs and the inflammatory immune response by showing that transfection of a constitutively activated TLR activated the NF-κB pathway [5]. Following these experimental studies, numerous clinical studies have confirmed the crucial role of TLR in the immune-inflammatory response induced by infections [6,7]. Moreover, strong associations between polymorphisms and mutations in TLR family members or downstream proteins and increased susceptibility and severity to infections have been reported in humans [8-12].

Such findings provided a strong rationale for the development of new therapeutic approaches based on the modulation of the dysregulated immune response responsible for organ failures during sepsis. However, experimental animal models do not fully elucidated the clinical conditions they aim to mimic, and both scientists and physicians have raised several criticisms on the relevance of pre-clinical experimental research based on animal models and its translation to human pathology. Some shortcomings are directly related to the type of animal models of sepsis. Thus, endotoxinic shock has often been used as a surrogate of septic shock, and experimental immunomodulatory drugs have most often been administrated prior to or concomitant to the infectious insult [13]. In addition, experimental outcomes related to a given intervention may not be consistent across different models. For instance, attempts of tumor necrosis factor alpha (TNFα) neutralization in experimental models resulted in discrepant results, either beneficial or detrimental, depending on the type of infectious insult [14]. This skepticism was further increased by the accumulation of negative human trials of immunomodulatory drugs that had previously shown dramatic benefits in experimental studies (Table 1). Despite promising results in animal models, all newly developed drugs such as anti-TNFα antibodies [15] or the TLR4-antagonist eritoran [16] failed to improve survival to human severe sepsis and septic shock and sometimes even worsened the patient’s prognosis when using nitric oxide synthase inhibitor or TNFα receptor:Fc fusion protein [17,18].

**Table 1:** **Examples of negative human therapeutic trials targeting successive inflammatory pathways involved in the pathophysiology of sepsis**

**Steps of the inflammatory response**	**Target**	**Treatment**	**Included population**	**References**
Bacterial components	Bacterial membrane	Recombinant bactericidal protein (rBPI21)	Meningococcal sepsis (*n* = 393)	Levin et al. Lancet 2000 [76]
	LPS	Murine anti-endotoxin antibody	Severe sepsis (*n* = 1,090)	Angus et al. JAMA 2000 [77]
Receptors	TLR-4	Lipid antagonist of MD2-TLR4	Severe sepsis (*n* = 1,961)	Opal et al. JAMA 2013 [78]
Intracellular signaling	Inflammatory pathways	Methylprednisolone (high doses)	Severe sepsis (*n* = 382)	Bone et al. N Engl J Med 1987 [79]
Cytokine production	TNF-α	Soluble TNF receptor	Septic shock (*n* = 141)	Fisher et al. N Engl J Med 1996 [18]
	IL-1	IL-1 receptor antagonist	Severe sepsis (*n* = 696)	Opal et al. Crit Care Med 1997 [80]
Enzyme activation	Phospholipase	PAF inhibitor BB-882	Severe sepsis (*n* = 152)	Vincent et al. Crit Care Med 2000 [81]
Oxidative stress	Reactive oxygen species	Selenium	Severe sepsis (*n* = 150)	Valenta et al. Intensive Care Med 2011 [82]

LPS, lipopolysaccharide; TLR4, Toll-like receptor 4; TNF-α, tumor necrosis factor alpha; IL, interleukin; PAF, platelet activating factor.

Whether data observed in animals are really relevant to the human conditions is certainly a major concern in medical research. Discrepancies between species have been recently highlighted in a study that explored the variation of genomic response (>4,000 genes) in human and murine blood-derived leucocytes following several types of injuries (trauma, burn, infection) including stimulation by lipopolysaccharide (LPS) [19]. Changes in the gene expression profile, assessed at different time points after endotoxemia, displayed a very low correlation between human and mouse genomic responses thereby raising an important concern about the usefulness of mouse models in sepsis research. However, we keep thinking that animal models remain irreplaceable tools for advances in the field of critical care medicine. The clear demonstration of ventilator-induced lung injury in experimental animal models directly translated into improvements in clinical care through protective ventilation that is now applied in nearly all patients undergoing invasive mechanical ventilation [20,21]. Should we continue to use animal models for the purpose of drug development, the reproducibility of any improvement in outcome should be addressed more extensively in animals with different genetic background and underlying conditions such as age or co-morbidities and by using various infection models. Beyond the imperfect animal models of critical illnesses, the failure of therapeutic clinical trials may be intrinsic to their design as they were aimed to treat a syndrome rather than a disease and thereby did not take into account the major heterogeneity of critically ill patients [22].

### Sepsis-induced immunosuppression: from human to animal and back to human

The concept of sepsis-induced immunosuppression represents a striking example of the potential of translational research in critical care medicine. It illustrates the full virtuous circle of research performed from man to mouse and from mouse back to man, with the ultimate purpose of developing novel therapeutic strategies.

Severe sepsis and septic shock remain associated with high mortality rate despite advances in the management of infection and organ failures [23,24]. It is noteworthy that the majority of patients now survive the initial septic insult but then become highly susceptible to secondary ICU-acquired infections [25,26]. As mentioned above, anti-inflammatory clinical trials applied at the initial phase of sepsis failed to tackle the devastating effects of sepsis and to translate into improved survival. This suggests that the initial pathophysiological hypothesis may have occulted some alternative immune changes. Furthermore, the classic paradigm of sepsis was dramatically challenged in the late 1990s by Richard Hotchkiss’s pioneering works, in which post-mortem biopsies from patients deceased from sepsis revealed a major loss of immune cells within lymphoid organs [27]. These findings were reminiscent of under-recognized historical observations that were already suggestive of acquired immunosuppression in septic patients, such as unreactive skin testing owing to defective type IV hypersensitivity or altered *in vitro* cytokine production by monocytes, both associated with increased mortality [28-30].

On this basis, the conception of the immune pathophysiology of sepsis dramatically evolved and now encompasses both an initial tremendous systemic inflammatory response responsible for organ failures and a compensatory anti-inflammatory response with multiple immune defects that may result in complex immunosuppression in some patients [31]. The main features of sepsis-induced immunosuppression have been recently reviewed [32]. It is noteworthy that both innate and adaptive arms of the immune response are seriously impaired. Since most patients survive the first days of sepsis, it has been postulated that acquired immune alterations may directly participate in a worse outcome through the inability to clear the initial infectious focus and/or decreased resistance to ICU-acquired infections. This hypothesis has been investigated in patients in whom acquired defects in most circulating immune cells have been strongly associated with the outcome and the development of nosocomial infections. Importantly, it is now clear that the time course of immune defects during the ICU stay is more accurate than the initial depth of biological abnormalities in predicting the development of ICU-acquired infections [33-35].

A reliable appraisal of the role of sepsis-induced immunosuppression in clinical settings is limited by the multiple confounding risk factors of nosocomial infections. This prompted the development of experimental research programs aimed to mimic the common clinical situation of severe sepsis followed by secondary infections in rodent double-hit models. The increased susceptibility of post-septic animals to subsequent infectious insults either to weakly virulent pathogens or to low pathogen loads known to be innocuous in immune-competent animals provided a firm proof-of-concept of sepsis-induced immune suppression [36,37]. These animal models allowed the characterization of immune cells’ behavior within organs, highlighted the aberrant immune response underlying the increased susceptibility of septic host towards a secondary infectious insult, and are currently used to decipher the regulatory mechanisms of sepsis-induced immunosuppression. Such models are irreplaceable tools to address the efficacy of potential immunomodulatory therapeutic interventions such as cytokines (interleukin-7 (IL-7), interferon-γ (IFN-γ)), growth factors (granulocyte-monocyte colony-stimulating factor (GM-CSF)), or cell therapy aimed to restore an appropriate immune response in post-septic animals. This experimental step represents the preclinical requisite rationale before attempting immunomodulation in septic patients.

The accumulation of clinical and experimental evidence has fed the emerging idea that restoring immune competency might represent an innovative therapeutic target in patients during the immunosuppressive phase of sepsis, in order to improve survival and/or to prevent nosocomial infections (Table 2). Attempts of immune-stimulating therapeutic interventions based on some biomarkers of immunosuppression have been performed. The first results have been promising with IFN-γ or GM-CSF aimed to reverse monocyte deactivation [38,39], and new candidates are emerging from the field of cancer such as IL-7 or anti-PD-1-related molecules [32,40]. Such biomarker-guided therapeutic interventions may represent a milestone in the development of a personalized medicine in the ICU which is probably more relevant to the heterogeneity of critically ill patients. Thanks to biotechnological advances, some tools have been developed to monitor patients’ immune functions in a more reproductive manner and identify those likely to benefit from immune stimulation while minimizing the potential risk of harmful side effects [41,42]. Both identification of the most accurate therapeutic target and reliable immunomonitoring are keys to the eventual success of new therapeutic interventions such as GM-CSF and IL-7 that are currently evaluated in clinical trials.

**Table 2:** **Therapeutic targets and potential treatments of sepsis-induced immunosuppression**

**Biomarker**	**Potential therapeutics**	**Clinical data**	**Comments**
Monocyte deactivation	GM-CSF	Yes	Restoration of HLA-DR expression [39,83]
			Clearance of uncontrolled infections [83]
			Reduced duration of mechanical ventilation [39]
	IFN-γ	Yes	Restoration of HLA-DR expression [38]
Apoptosis of immune cells	Anti-apoptotic cytokines	No	
	Caspase inhibitors	No	
	Death-receptor antagonists	No	
Increased Tregs	Anti-Tregs antibodies	No	
Depletion/deactivation of dendritic cell	Flt3-L	No	
	TLR-agonists	No	
T cell exhaustion	IL-7	Yes	*Ex vivo* restoration of lymphocyte functions [40]
	Thymosin-α	Yes	Improved survival in sepsis due to carbapenem-resistant bacteria (in association with ulinastatin) [84]
	IL-15	No	
Upregulated expression of co-inhibitory receptors	Monoclonal antibodies:		
	Anti-PD1/PDL1	No	
	Anti-CTLA-4	No	
	Anti-BTLA	No	

GM-CSF, granulocyte-monocyte colony-stimulating factor; IFN-γ, interferon-γ; HLA-DR, human leukocyte antigen-DR; Tregs, T regulatory lymphocytes; Flt3-L, ligand of the fms-like tyrosine kinase 3; TLR, Toll-like receptor; IL, interleukin; PD1, programmed death 1; PDL1, programmed death ligand 1; CTLA-4, cytotoxic T-lymphocyte-associated protein 4; BTLA, B and T lymphocyte attenuator.

### Critical illness-associated neuromyopathy: from human to human

Studies of the mechanisms of critical illness-associated neuromyopathy (CINM) illustrate how a human-to-human translational research program can be effective for investigating pathological disorders and eventually improving care [43-46]. CINM, also known as ICU-acquired paresis or weakness, is a frequent and severe complication of critical illness, associated with increased mortality, delayed weaning from the ventilator, and long-term functional disability [47-50]. CINM is clinically characterized by a bilateral and symmetric peripheral motor deficit, sparing cranial nerves [48]. It is most often related to myopathy that may be associated with sensory motor axonal polyneuropathy which significantly worsens the functional outcome [51].

The pathophysiology of CINM is complex and encompasses various and intertwined mechanisms at different stages of critical illness, from insult to recovery. Observational studies have enabled to identify risk factors of CINM related both to the critical illness and to the ICU management, giving clues to underlying pathogenic mechanisms. The main factors contributing to CINM appear to be an intense systemic inflammatory response, unloading, malnutrition and endocrine-related anabolism/catabolism imbalance, drugs (such as steroids or neuromuscular blocking agents), and electrolyte disturbances [51,52]. Neurophysiological and histological investigations have been of great help for identifying the pathogenic mechanisms of CINM. The diagnostic and prognostic values of electroneuromyogram are clearly established [48]. The observation that muscle and nerve inexcitability was an early phenomenon predictive of muscle weakness raised the hypothesis of a channel dysfunction that was subsequently confirmed by alternative experiments [53]. The interest of muscle biopsy has been remarkably demonstrated by Bradley et al. [54], by Puthucheary et al. [46], and by Hermans et al. [43], who thereby assessed the role of mitochondrial dysfunction, proteolysis, and autophagy, respectively. However, the routine applicability of muscle biopsy at the bedside remains limited. Furthermore, the diaphragm is the main respiratory muscle and is liable to early-onset injury processes in ventilated patients but is hardly accessible for biopsy [55,56]. Some non-invasive imaging solutions have become of interest in order to address muscular dysfunction in the ICU. Ultrasound imaging is a simple and convenient tool to assess and follow up muscle atrophy. Furthermore, magnetic resonance (MR), especially ^31^P, ^1^H, or ^13^C spectroscopy, allows studying the biomechanical and functional properties of skeletal muscle *in vivo* [57]. Constraints and risks of nerve biopsy limit the investigations in the pathophysiology of the critical illness-associated neuropathy. Skin biopsy might be a convenient alternative to nerve biopsy in order to study small-fiber neuropathy, while advanced electrophysiological explorations may assess sodium channel or synaptic dysfunctions.

Hence, a ‘human-to-human’ translational research was able to provide a comprehensive assessment of the clinical entity of CINM and resulted in very significant insights into the underlying pathophysiological mechanisms. Recognition of CINM is based on clinical examination, while imaging and neurophysiological explorations can contribute to the prognosis and follow-up of the disorder. However, current therapeutic interventions for CINM remain quite limited, based on reducing the exposure to risk factors, sustaining appropriate nutritive supply during the ICU stay and early rehabilitation [58]. Beyond the informative but obviously limited translational research performed in humans, some relevant animal models, mainly based on experimental sepsis, are being developed in order to further decipher the cellular and molecular mechanisms of CINM and to allow the preclinical evaluation of new therapeutics [53].

### Monitoring of the microcirculation: *in vivo* technological assay

Microcirculatory perfusion deals with the circulation of blood through the small arteries, capillaries, and venules of different tissues. This complex microvascular network is responsible for regulation of vascular tone, adaptation of tissue oxygenation to local metabolic demand, exchange of nutrients and waste products between blood and cells, and regulation of immune response [59]. Experimental studies have shown that impairment in macro-hemodynamics does not fully account for the mismatch between oxygen demand and supply in sepsis and that pathological arterio-venous shunt formation may also contribute to flow misdistribution and eventual impairment in oxygen delivery [60]. *In vivo* video microscopy revealed intestinal mucosal hypoperfusion during murine endotoxemia. Most importantly, a decrease in arteriolar diameters and in tissue blood flow occurred in both hypotensive and normotensive animals [61]. Using intravital microscopy in a rodent model of cecal ligation and puncture, Piper et al. demonstrated that similar microcirculatory alterations occurred within skeletal muscles [62]. The increased proportion of non-perfused capillaries was associated with a decrease in capillary-venular oxygen saturation of hemoglobin and an increased capillary oxygen extraction, as assessed by spectrophotometric functional imaging system [63]. As such, microvascular alterations have been advocated as one of the main determinants in the pathogenesis of sepsis-related organ dysfunction through multiple mechanisms, including endothelial dysfunction, impaired inter-cellular communication, altered glycocalyx, adhesion and rolling of white blood cells and platelets, and altered red blood cell deformability [60].

**Figure 1:**
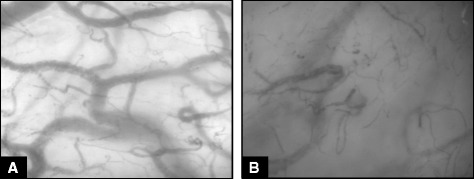
Sublingual microcirculation in a healthy volunteer **(A)** and in a patient with septic shock **(B)**. The density of small vessels is significantly reduced during septic shock along with areas of different capillary density, the so-called microvascular heterogeneity.

The evaluation of microcirculation in human pathology started only later, when the side-stream dark field (SDF) imaging technique became available at the bedside [59]. Almost ten years ago, altered microcirculatory perfusion was thus demonstrated in patients with severe sepsis and septic shock when compared to healthy volunteers and other non-septic critically ill patients. Sepsis-induced microvascular alterations were assessed in the sublingual area and were characterized by highly heterogeneous flow and decreased capillary density with increased numbers of stopped-flow and intermittent-flow capillaries (Figure 1) [64]. More severe and prolonged impairment in microcirculation was observed in non-survivors [64-66]. Importantly, these alterations could be reversed by therapeutic interventions such as early fluid loading or intravenous administration of nitroglycerin, dobutamine, or activated protein C [67-70]. In addition, topical application of acetylcholine could restore normal microvascular perfusion, suggesting that thrombus formation into the capillaries was not an essential pathophysiological mechanism involved in these alterations [64].

However, most human studies only evaluated the sublingual area that may not fully encompass microvascular flow or changes in tissue oxygenation and metabolism within organs. Recent experimental studies have contributed to better understand the role of microcirculation in organ dysfunction during sepsis. In a porcine model of cholangitis-induced septic shock, Verdant et al. showed that severity and time course of microcirculatory changes were similar in the gut and the sublingual areas, suggesting that this latter region would be appropriate to monitor systemic microcirculatory alterations [71]. Microcirculation was altered even in the brain cortex during experimental sepsis and such changes were independent from systemic hemodynamics [72]. Also, impaired cerebral microcirculation was associated with a progressive reduction in brain oxygenation and, in association with the development of severe hypotension, was responsible for anaerobic metabolism [73]. Impairment in microcirculation has also been observed in the liver of septic rats and was improved by hemodynamic resuscitation with crystalloid solutions, but not with gelatines [74]. Interestingly, sepsis did not alter renal cortical microcirculation in the early phase, while norepinephrine induced renal vasoconstriction [75].

Developments in the monitoring of the microcirculation illustrate how sophisticated technological tools may improve our comprehension of pathophysiology, as well as their limits to implementation into routine clinical practice. Altogether, the aforementioned experimental findings clearly demonstrated that microvascular dysfunction occurs during sepsis in several peripheral organs. Given the heterogeneous aspect of microcirculatory perfusion and the mechanisms involved in the development of such dysfunctions, it is unlikely that classical interventions aimed to restore global hemodynamics might result in significant improvement in microvascular flow. Nevertheless, we are still lacking some appropriate devices for clinical settings, which should ideally combine assessment of microcirculation and tissue oxygenation. Future research should be directed towards improvement in technologies that may increase the accuracy of microcirculatory assessment in septic patients. Most importantly, it remains unknown whether assessment of microcirculation and changes induced by therapeutic interventions may lead to improved management of critically ill patients. A number of questions remain to be supported by robust clinical and experimental data, including the type of therapeutic interventions and their optimal timing after hemodynamic stabilization, and the relevant target goals in capillary flow or tissue oxygenation that should then be achieved.

## Conclusions

### Benefits and usefulness of translational research?

From a clinical point of view, translational research has produced contrasted results in critical care medicine. It clearly offered major advances in the understanding of the pathophysiology of sepsis, and animal models have underscored the importance of early antibiotic treatment and aggressive resuscitation that are now commonly implemented during the so-called golden hours of clinical management. However, advances in the comprehension of the immunopathology of sepsis that relied on imperfect animal models failed to translate into successful innovative therapeutic approaches in humans. Nevertheless, we learned important lessons from negative trials, and focusing on a single therapeutic target can now be viewed as simplistic with respect to the complexity of the immune response. In order to sustain innovative approaches in critically ill patients, the French Society of Intensive Care is committed to promote translational research in critical care medicine.
